# Impact of fine-needle aspiration cytology in thyroidectomy extent and associated surgical morbidity in thyroid cancer

**DOI:** 10.1007/s00423-024-03258-3

**Published:** 2024-02-19

**Authors:** Patrik Lind, Erik Nordenström, Lars Johansson, Göran Wallin, Kosmas Daskalakis

**Affiliations:** 1 Anesthesiology Department, Skellefteå Hospital, Skellefteå, Sweden; 2 Department of Surgery, Faculty of Medicine and Health, Örebro University, 70185 Örebro, Sweden; 3 Department of Surgery, Skåne University Hospital, Lund, Sweden; 4 Department of Clinical Sciences, Lund University, Lund, Sweden; 5 Department of Public Health and Clinical Medicine, Skellefteå Research Unit, Umeå University, 901 81 Umeå, Sweden; 6 Second Department of Surgery, “Korgialenio-Benakio,” Red Cross General Hospital, Athens, Greece

**Keywords:** Fine-needle aspiration cytology, Thyroid cancer, Surgical morbidity

## Abstract

**Purpose:**

To assess the impact of fine-needle aspiration cytology (FNAC) in the extent of surgery in patients with thyroid cancer (TC) and the associated surgical morbidity in primary and completion setting.

**Methods:**

A Swedish nationwide cohort of patients having surgery for TC (*n* = 2519) from the Scandinavian Quality Register for Thyroid, Parathyroid and Adrenal surgery between 2004 and 2013 was obtained. Data was validated through scrutinizing FNAC and histology reports.

**Results:**

Among the 2519 cases operated for TC, the diagnosis was substantiated and validated through the histology report in 2332 cases (92.6%). Among these, 1679 patients (72%) were female, and the median age at TC diagnosis was 52.3 years (range 18–94.6). Less than total thyroidectomy (LTT) was undertaken in 944 whereas total thyroidectomy (TT) in 1388 cases. The intermediate FNAC categories of atypia of undetermined significance/follicular lesion of undetermined significance (AUS/ FLUS), as well as suspicion for follicular neoplasm (SFN) lesions were more often encountered in LTT (*n* = 314, 33.3%) than TT (*n* = 63, 4.6%), whereas FNACs suspicion for malignancy and/or malignancy were overrepresented in TT (*n* = 963, 69.4%). Completion thyroidectomies were undertaken in 553 patients out of 944 that initially had LTT. In 201 cases with cancer lesions > 1 cm, other than FTC (Follicular TC)/ HTC (Hürthle cell TC) subjected to primary LTT, inadequate procedures were undertaken in 81 due to absent, Bethesda I or II FNAC categories, preoperatively. Complications at completion of surgery in this particular setting were 0.5% for RLN palsy (*n* = 1) and 1% (*n* = 2) for hypoparathyroidism 6 months postoperatively. The overall postoperative complication rate was higher in primary TT vs. LTT for RLN palsy (4.8% [*n* = 67] vs. 2.4% [*n* = 23]; *p* = 0.003) and permanent hypoparathyroidism (6.8% [*n* = 95] vs. 0.8% [*n* = 8]; *p* < 0.0001).

**Conclusions:**

FNAC results appear to affect surgical planning in TC as intermediate FNAC categories lead more often to LTT. Overall, inadequate procedures necessitating completion surgery are encountered in up to 15% of TC patients subjected to LTT due to absent, inconclusive, or misleading FNAC, preoperatively. However, completion of thyroidectomy in this setting did not yield significant surgical morbidity. Primary LTT is a safer primary approach compared to TT in respect of RLN palsy and permanent hypoparathyroidism complication rates; therefore, primary TT should probably be reserved for lesions > 1 cm or even larger with suspicion for malignancy or malignant FNAC.

## Introduction

Thyroid cancer (TC) is the most common endocrine malignancy with increasing incidence over the last decades [1–3]. Despite that the observed increase in TC incidence can be partly attributed to early detection due to the widespread use of ultrasonography and fine-needle aspiration cytology (FNAC), the evident increase in the incidence of palpable TC as well as the rising mortality rates in advanced disease stages imply a true increase in the occurrence of TC [1, 3–5].

FNAC remains the first-line tool with satisfactory results in the preoperative diagnosis of thyroid nodules. Nevertheless, ultrasonographic assessment of thyroid lesions and its utilization in FNAC guidance have proven very helpful in establishing the correct diagnosis [6]. In the past few years, standardization of diagnostic terminology following the Bethesda System for Reporting Thyroid Cytology has been adopted in clinical practice worldwide, providing an evidence-based approach for the management of thyroid nodules [7, 8].

Differences in the extent of surgery in respect of preoperative FNAC findings, in particular, the use of total thyroidectomy (TT) vs. less than total thyroidectomy procedures (LTT), mainly lobectomies, are multifaceted and generally depend on patient- and tumour-related factors discussed at different regional multidisciplinary tumour boards but generally dictated by the Swedish thyroid cancer program guidelines [9]. In addition, certain Bethesda categories, particularly the indeterminate categories of atypia of undetermined significance/follicular lesion of undetermined significance (AUS/ FLUS), as well as suspicion for follicular neoplasm lesions (SFN) pose significant diagnostic and therapeutic difficulties, as they demonstrate a broad range of histologic outcomes often necessitating primary diagnostic LTTs without lymph node dissection (LND) followed by completion surgery in cases of TC histopathology of the surgical specimen [10–13]. However, in cases of inconclusive or benign FNAC, follow-up with repeated ultrasonographic testing and/or repeated FNAC is generally recommended [14]. Nevertheless, the extent of surgery may affect patient outcomes both in terms of survival and recurrence rates, as well as in terms of postoperative surgical morbidity [15].

The aim of the present study was to assess the impact of preoperative FNAC in the surgical management of TC patients and in respect of the rates of malignancy in cytology reports and final histology. In particular, we aimed to assess the clinical implications of FNAC in the extent of surgery, including the rate of absent or misleading FNAC leading to inadequate procedures, as well as complications associated with completion surgery in the latter subset.

## Material and methods

The study was approved by the regional ethical committee in Uppsala and the Swedish Ethical Review Authority (2012/055 and 2020–05783). Data were primarily collected from the Scandinavian Quality Register for Thyroid, Parathyroid and Adrenal Surgery (SQRTPA), https://sqrtpa.se and subsequently validated through scrutinizing FNAC and histology reports across 37 hospitals in Sweden. SQRTPA contains data on patient demographics, preoperative, as well as operative and postoperative data, histopathological information, and follow‐up information at 6 weeks and 6 to 9 months after surgery; with a 90% coverage for thyroid procedures at a national level. Data on adult patients (> 18 years) with a postoperative histopathological diagnosis of TC operated upon between 1 January 2004 and 31 December 2013 were extracted from the SQRTPA. Subsequently, registry data was validated through scrutinizing FNAC and histology reports by the author PL, who visited all hospitals performing thyroid surgery in Sweden. In particular, a questionnaire for each patient was filled in by surgeons at all participating clinics connected to the SQRTPA registry during the inclusion period of the study, and also, a second questionnaire for all included patients in this study was sent to the attending surgeons in the SQRTPA participating departments, and pertinent data was extracted by PL. Only patients with available histopathological reports and definite histopathological diagnosis of TC on primary thyroid surgery were included. Patients subjected to completion surgery during the study period without available data from the primary procedure (histopathology and preoperative cytology) e.g. cases primarily operated prior to the inclusion period with incomplete data were excluded. In addition, apart from completion procedures, cases reported as TC in SQRTPA, with a benign histology at primary operation were considered misclassifications and therefore excluded from the present study. Information about postoperative complications, such as recurrent laryngeal nerve palsy and postoperative hypoparathyroidism is self‐reported to the SQRTPA by surgeons at involved centres and was extracted solely by the SQRTPA.

Cytological diagnoses were categorized into five groups: non-diagnostic, benign, AUS/FLUS and follicular neoplasm, suspicion for malignancy and malignant FNAC. As part of the present cytological data was obtained prior to the widespread Bethesda adoption and its introduction to the Swedish clinical practice, we tried to recode all cytological reports following the Bethesda system. However, since it requires histologic evidence of capsular/vascular invasion to distinguish follicular adenoma from follicular carcinoma, and nodules selected for surgery may have other clinical or ultrasonographic features that increase suspicion, we did not attempt to distinguish AUS/FLUS and follicular neoplasm categories.

In respect of surgical indications in this cohort during the study period, all intermediate FNAC categories i.e. patients with AUS/FLUS and SFN results were subjected to surgery, mainly LTT (diagnostic lobectomies) and were not considered for repeat biopsy and/or additional adjunct testing like molecular testing, which was not available at that time period. In addition, although FNAC in lesions < 1 cm was not generally recommended, positive for malignancy FNACs commonly resulted in referrals for surgery.

With regard to the implications of FNAC in the extent of surgery, we investigated the frequency of each FNAC category in respect of the type of thyroid surgery (TT vs. LTT) and the FNAC findings in cases of LND. Moreover, we calculated the rate of FNAC misdiagnosis in TC cases i.e. non-diagnostic, benign or even intermediate FNAC in certain TC subsets leading to inadequate procedures and complications associated with completion surgery. A 10-mm size cutoff was applied to assess the impact of FNAC on the extent of surgery with regard to microcarcinomas.

### Statistical analysis

Continuous variables are reported as median with range or mean with standard deviation (SD), as indicated by the data distribution. Statistical analyses were performed using SPSS v25 (IBM Corp., Armonk, NY). Comparative analyses were performed with the Pearson χ^2^ test for categorical variables. All *p*-values were predetermined to be two-sided, with the level of significance set at *p* < 0.05.

## Results

**Fig. 1:**
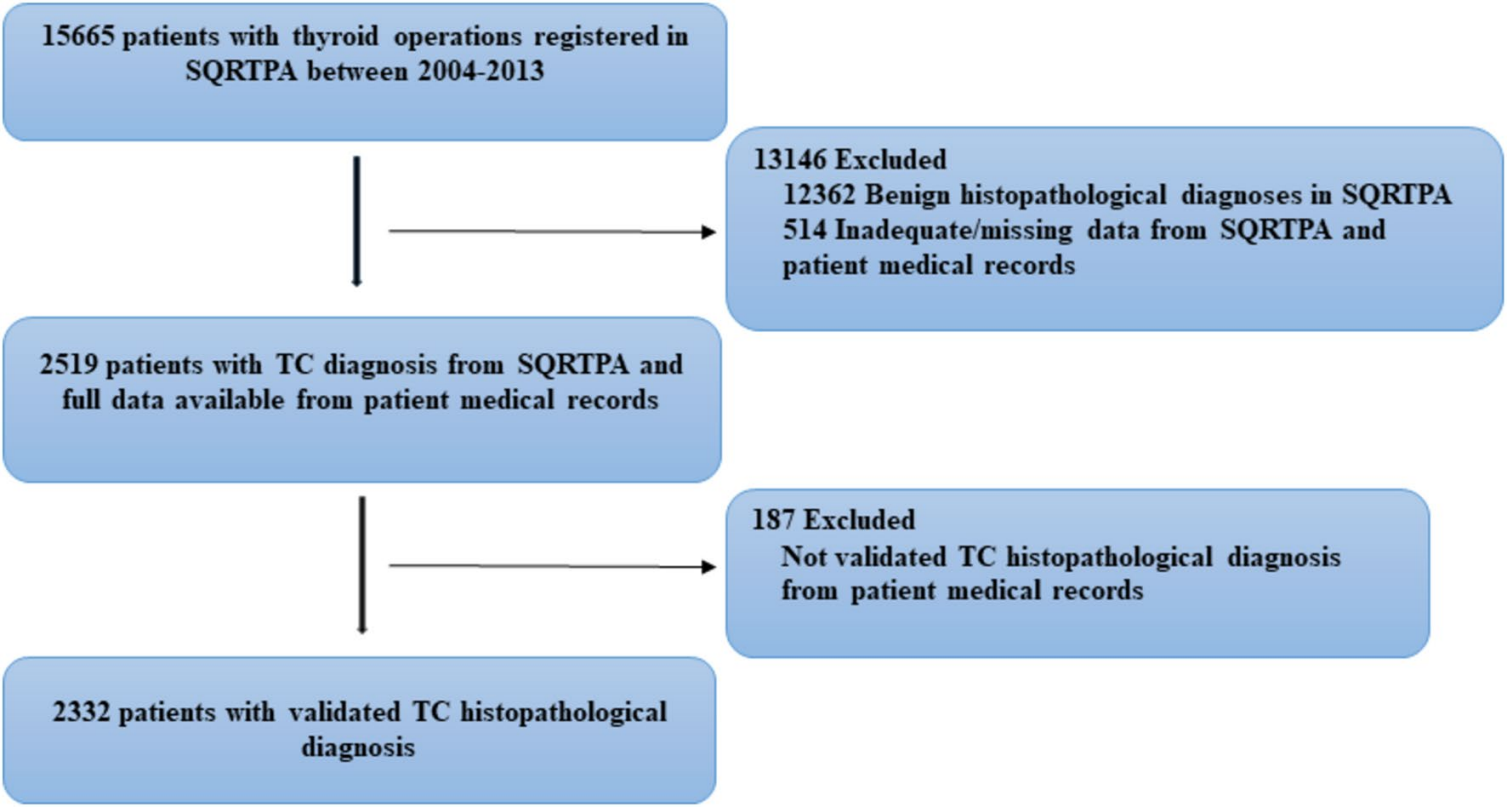
Study flow diagram. Abbreviations: TC, thyroid cancer; SQRTPA, Scandinavian Quality Register for Thyroid, Parathyroid and Adrenal surgery

**Fig. 2:**
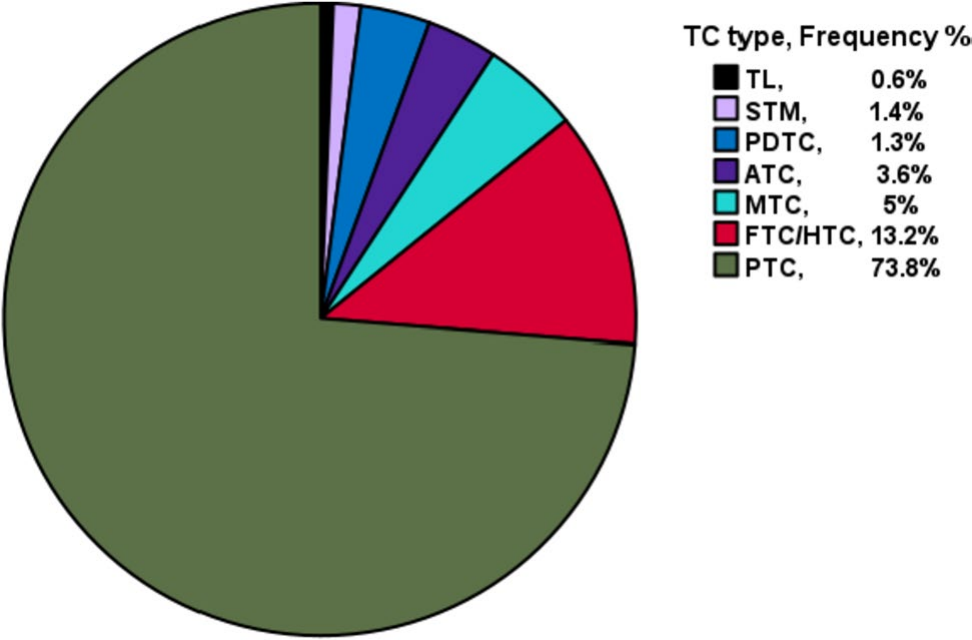
Distribution of different thyroid cancer types in Sweden. Abbreviations: TC, thyroid cancer; TL, thyroid lymphoma; STM, secondary thyroid malignancies; PDTC, poorly-differentiated thyroid carcinoma; ATC, anaplastic thyroid cancer; MTC, medullary thyroid cancer; FTC, follicular thyroid cancer; HTC, Hürthle-cell thyroid cancer; PTC, papillary thyroid cancer

Among the 2519 cases operated for TC in SQRTPA, we included 2332 cases (92.6%) with validated TC histopathological diagnosis from the primary operation. The study flow is given in Fig. 1. Among these, 1679 patients (72%) were female, and the median age at TC diagnosis was 52.3 years (range 18–94.6). The majority of cases were well-differentiated TC (WDTC), including mainly PTC (*n* = 1722; 73.8%), followed by minimally invasive follicular thyroid cancer/Hürthle cell thyroid cancer (FTC/HTC; *n* = 280; 12%) and widely invasive FTC/HTC (*n* = 51; 2.2%). We encountered 297 non-WDTC cases (12.7%), including poorly differentiated TC (*n* = 31; 1.3%), MTC (*n* = 116; 5%), ATC (*n* = 84, 3.6%), thyroid lymphoma (*n* = 15, 0.6%) and finally secondary thyroid malignancies in 33 cases (1.4%). In Fig. 2, a pie-chart of TC types’ distribution in our nationwide cohort of TC patients during the 10-year period of the study is given.

With regards to the implications of FNAC in the extent of surgery, the frequency of each FNAC category in respect of the type of thyroid surgery (TT vs. LTT), as well as in cases of LND are shown in Table 1. LTT was undertaken in 944 cases (commonly lobectomy, *n* = 866), whereas TT was in 1388 cases. In 1238 cases of the present cohort, LND was undertaken (central LND [CLND, *n* = 831], lateral LND [LLND, *n* = 407]). The intermediate FNAC, categories of AUS/FLUS and SFN (*n* = 377) led more often to LTL procedures (*n* = 314, 83.3%) than TT (*n* = 63, 16.7%), whereas FNACs suspicion for malignancy and/or malignant FNACs (*n* = 1196) led to TTs (*n* = 963, 80.4%) rather than LTTs (*n* = 233, 19.6%). In addition, with respect to LNDs, FNACs suspicion for malignancy and/or malignant FNACs were present in 69.9% of CLNDs (*n* = 581) and 82.1% of LLNDs (*n* = 334, Table 1). Importantly, microcancer cases in the present cohort were accompanied with LND in 194/571 cases (34%, 121 CLND and 73 LLND), suggesting clinically significant microcarcinomas, often with locoregional LN involvement.

**Table 1:** Operative procedures with preoperative FNAC findings correlation from the Scandinavian Quality Register for Thyroid, Parathyroid and Adrenal Surgery between 2004 and 2013

Type of surgery	Cytology findings					
	*FNAC not performed*	Inconclusive FNAC	Benign FNAC	AUS, FLUS or follicular neoplasm	FNAC suspicion for	Malignant FNAC
Total thyroidectomy (*n* = 1388)	230 (16.5%)	13 (0.9%)	119 (8.6)	63 (4.6%)	91 (6.6%)	872 (62.8%)
Less than total thyroidectomy* (*n* = 944)	132 (14%)	28 (3%)	237 (25.1%)	314 (33.3%)	127 (13.5%)	106 (11.2%)
Central lymph node dissections (*n* = 831)	70 (8.4%)	7 (0.8%)	38 (4.6%)	135 (16.2%)	87 (10.5%)	494 (59.4%)
Lateral lymph node dissections (*n* = 407)	42 (10.3%)	5 (1.2%)	14 (3.4%)	12 (2.9%)	15 (3.7%)	319 (78.4%)

*Less than total thyroidectomies included lobectomies (*n* = 866), isthmus resections (*n* = 23), subtotal thyroidectomies (*n* = 31) and other resections (*n* = 24)

Abbreviations: *FNAC* fine-needle aspiration cytology, *AUS* atypia of undetermined significance, *FLUS* follicular lesion of undetermined significance

Completion thyroidectomies were undertaken in 553 patients out of 944 that initially had LTTs. Importantly, in a subset of 184 patients that were subjected to completion surgery, we only had data during the inclusion period on the primary procedure. On the other hand, patients with available data only on completion and not the primary procedure were not primarily included in the study cohort. Among the remaining 369 patients that were subjected to completion procedures and had available data both on primary and completion surgery, lesions > 1 cm in the primary operation were encountered in 304 cases (82.4%), lesions < 1 cm in 44 completion procedures, whereas 21 patients had no available data on tumour size. Among these 304 cases with tumours > 1 cm that were subjected to completion, in the primary preoperative setting, FNAC was not performed at all in 12 (3.9%); in 11 (3.6%), it was inconclusive; in 77 (25.3%) benign, in 138 (45.4) exhibited AUS/FLUS or SFN; in 55 (18.1%) FNAC, it was suspicion for malignancy, and finally in 11 (3.6%), FNAC was malignant.

In further analysis, when also excluding cases of diagnostic LTTs for lesions > 1 cm that turned out to be FTC and/or HTC > 1 cm (*n* = 102), in the remaining 202 cases, in the primary preoperative setting, FNAC was not performed at all in 5 (2.5%); in 10 (4.9%), it was inconclusive, in 66 (32.4%) benign, in 65 (31.9) exhibited AUS/FLUS or SFN; in 45 (22.1%), FNAC was suspicion for malignancy and in 11 (5.4%) malignant. Thus, in 202 cases with cancer lesions > 1 cm, other than FTC and HTC subjected to primary LTT, inadequate procedures were undertaken in 81 (40%) due to absent, inconclusive or misleading FNAC, whereas in 56 (27.5%), inadequate procedures were undertaken despite suspicion for malignancy or malignant FNAC. The rate of FNAC misdiagnosis in this subset was as high as 32%, when excluding cases that FNAC was not performed at all and non-diagnostic FNACs. Overall, in respect of final histopathology findings, FNAC has led to 81 completion thyroidectomies out of a total of 553 completion cases (15%) due to absent, inconclusive or benign FNACs. These LTTs could have been avoided if FNAC had been performed initially or repeatedly as necessary and upfront TT had been applied.

In the more difficult to diagnose preoperatively subset of patients with minimally-invasive FTC or HTC > 1 cm (*n* = 255), 180 patients (70.6%) were subjected to LTT (mainly diagnostic lobectomy, *n* = 171; 67.1%), whereas 75 patients (29.4%) had primarily TT. In these 180 patients with minimally invasive FTC or HTC > 1 cm that were subjected to LTT, FNAC was not performed at all in 12 (6.7%), in 32 (17.8%) it was benign, in 109 (60.6%) exhibited AUS/FLUS or SFN, in 20 (11.1%) FNAC was suspicion for malignancy, and in 6 (3.3%), it was malignant. Thus, the intermediate FNAC categories in LTTs were encountered more often in minimally invasive FTC or HTC > 1 cm (60.6%), as compared to non-FTC or HTC cases > 1 cm (31.9%).

In respect of nodal dissections, further analysis of our data revealed that 266 CLNDs were performed in primary LTTs (*n* = 944) and among these, 115 (43.5%) had Bethesda III and IV categories. Within the subset of MTC (*n* = 116 cases), 93 patients underwent nodal dissections. In 18 out of these 93 cases (19.4%), FNAC was not performed at all, and nodal dissections were performed due to high calcitonin levels and a suspected MTC diagnosis.

Complications at completion surgery in tumours > 1 cm, other than FTC or HTC initially subjected to LTT (*n* = 202), were 0.5% for recurrent laryngeal nerve (RLN) palsy (*n* = 1), 30.3% (*n* = 61) for early hypoparathyroidism at patient discharge, 2.5% (*n* = 5) for hypoparathyroidism 1 month postoperatively, 1% (*n* = 2) for hypoparathyroidism 6 months postoperatively, 1% (*n* = 2) for postoperative bleeding, whereas no cases of postoperative infection were reported in SQRTPA in this setting. On the other hand, the postoperative complication rate irrespective of tumour size and final histopathology (*n* = 1388) in our TC cohort was higher in primary TT (*n* = 1388) vs. LTT (*n* = 944) for RLN palsy (4.8% [*n* = 67] vs. 2.4% [*n* = 23]; *p* = 0.003), for early hypoparathyroidism at patient discharge (45% [*n* = 625] vs. 8.4% [*n* = 79]; *p* < 0.0001), for hypoparathyroidism 1 month postoperatively (11.5% [*n* = 160] vs. 1.3% [*n* = 12]; *p* < 0.0001), and for hypoparathyroidism 6 months postoperatively (6.8% [*n* = 95] vs. 0.8% [*n* = 8]; *p* < 0.0001). However, there was no significant difference between primary TT and LTT in postoperative bleeding (2.4% [*n* = 34] vs. 1.4% [*n* = 13]; *p* = 0.07) and postoperative infection rates (1.7% [*n* = 24] vs. 1.1% [*n* = 10]; *p* = 0.185) (Table 2). In view of the higher postoperative morbidity figures associated with TT, as compared to LTT both in terms of RLN palsy and permanent hypoparathyroidism, further data review revealed that LND was performed in 70% (*n* = 972) of patients in connection to primary TT (CLND = 611, LLND = 353, other = 8) in our dataset.

**Table 2:** Postoperative complication rates in respect of the extent of primary surgery and also in cases subjected to completion surgery*

Complications	Type of surgery			
	Primary LTT (*N* = 944)	Primary TT (*N* = 1388)	*Completion surgery (*N* = 202)	**P-value
RLN palsy	23 (2.4%)	67 (4.8%)	1 (0.5%)	**0.003**
Surgical wound infection	10 (1.1%)	24 (1.7%	0 (0%)	0.185
Bleeding	13 (1.4%)	34 (2.4%)	2 (1%)	0.07
Hypoparathyroidism at discharge	79 (8.4%)	625 (45%)	61 (30.3%)	** < 0.0001**
Hypoparathyroidism 1 month postop	12 (1.3%)	160 (11.5%)	5 (2.5%)	** < 0.0001**
Hypoparathyroidism 6 months postop	8 (0.8%)	95 (6.8%)	0	** < 0.0001**

*Completion surgery in tumours > 1 cm, other than FTC/HTC initially subjected to LTT

**Primary LTT vs. primary TT comparison

Statistically significant *P*-values are highlighted in bold

*LTT* less than total thyroidectomy, *TT* total thyroidectomy, *RLN* recurrent laryngeal nerve, *FTC* follicular thyroid cancer, *HTC* Hürthle-cell thyroid cancer

## Discussion

This study explored the implications of FNAC in the extent of thyroid surgery in 2332 TC cases across a 10-year period from the SQRTPA registry with nationwide validation and FNAC and histology correlation. The intermediate FNAC categories of AUS/ FLUS, as well as s SFN lesions (*n* = 377) led more often to LTTs (*n* = 314, 83.3%) that were mainly undertaken as a diagnostic procedure and were predominantly lobectomies. On the contrary, intermediate FNAC led less often to TT (*n* = 63, 16.7%). Furthermore, FNACs suspicion for malignancy and/or malignancy (*n* = 1198) were overrepresented in TTs (*n* = 963, 80.4%), as compared to those in patients subjected to LTTs (*n* = 235, 19.6%). Inadequate procedures in non-intermediate FNAC categories necessitating completion surgery are encountered in up to 40% of TC patients subjected to LTT for non-FTC/HTC lesions > 1 cm due to absent, inconclusive or misleading (benign) FNAC preoperatively; and overall, among 15% of all LTTs that led to completion.

TC distribution in the national registry ranged from WDTC to non-WDTC cases, as well as few secondary malignancies, and was mainly represented by PTC (*n* = 1722; 73.8%), followed by minimally invasive FTC/HTC cases (*n* = 280; 12%) in accordance with SEER database, and other registries [3, 16]. With regards to tumoural size, a significant proportion of tumours were < 1 cm in size (24.5%). FNAC utilization in microcarcinoma was limited, as 33.5% of cases were not subjected to FNAC. Importantly, microcarcinomas are probably underreported in the national registry, as they generally lack clinical significance [14, 17]. However, many micro-TC surgical cases in the present cohort were accompanied with nodal dissections (34%) often with node-positive disease, being therefore reported in the registry due to the disease stage and its clinical significance.

Previous American Thyroid Association (ATA) treatment guidelines for patients with well-differentiated TC [18] suggested LTT with lobectomy only for low-risk patients with microcarcinoma. However, lobectomy for pT1b and pT2 WDTC seems equivalent in term of survival and recurrence to TT [19, 20]. Therefore, according to the latest ATA recommendations [14], TT may be reserved for well-differentiated TC > 4 cm, cases of extrathyroidal invasion, and locoregional or distant stage disease. For lesions between 1 and 4 cm in size, either a LTT with lobectomy or a TT may be a valid option. However, the TC cases included herein during the inclusion period were treated following recommendations of regional multidisciplinary tumour boards, and in accordance with the Swedish thyroid cancer program guidelines that mainly suggested TT for lesions > 1 cm with suspicion for malignancy or malignant FNAC at that time. However, the reason for LTT could be multifactorial and not only determined by preoperative FNAC findings. Indeed, parameters such as thyroid volume (e.g. unilateral goiter), thyroid function (e.g. toxic adenoma) and specific ultrasound findings (EU-TIRADS categories < 4) may have affected the extent of the primary procedure with a more conservative procedure (LTT) planned beforehand in selected cases.

In our analysis, in the primary preoperative setting among 202 cases with cancer lesions > 1 cm other than FTC and/or HTC, inadequate procedures were undertaken in 40% due to absent, inconclusive or misleading (benign) FNAC, whereas in 27.5%, inadequate procedures were undertaken despite suspicion for malignancy or malignant FNAC. Interestingly, in 27.5% of these cases, FNAC was giving a correct positive result that was for unknown reasons not acted upon by the surgeon. This inconsistency may not be solely attributed to the fact that FNAC findings were not taken into consideration but could have been explained by factors such as patient comorbidities, age and patient preferences. Thus, in respect of final histopathology findings, FNAC has led to at least 81 completion thyroidectomies out of a total of 553 completion cases (15%) that could have been avoided if meticulous FNAC review with or without repeat FNAC had been undertaken, and upfront TT had been applied for patients with initially absent or Bethesda I and II FNAC categories. Although we acknowledge that factors other than preoperative FNAC findings may have also affected the extent of primary surgery, we feel that the absence of FNAC in TC lesions > 1 cm is clinically relevant, and if a meticulous preoperative assessment would have taken place including FNAC, this would probably change the initial approach of lobectomy (diagnostic or not) in many cases.

In 38 patients subjected to CLND (4.6%), the result of FNAC was benign, whereas in 70 patients (8.4%), FNAC was not performed at all. In another 135 patients (16.2%), CLND was performed in patients with Bethesda III and IV results. Importantly, within MTC (*n* = 116 cases), 93 patients underwent nodal dissections, and in 18 out of these 93 cases (19.4%), FNAC was not performed at all. This means that in 18 out of 70 CLNDs without preoperative FNAC (26%), this was undertaken due to high calcitonin levels and a suspected MTC diagnosis. In the remaining cases, the reasons could be multifaceted including clinical intraoperative suspicion of lymph node metastases in the central neck compartment. Nevertheless, our study includes many prophylactic CLNDs, an approach that is no longer recommended by ATA and Swedish national guidelines for patients with pT1 and pT2 PTC. Interestingly, further analysis of our data revealed that 266 CLNDs were performed in primary LTTs (*n* = 944), and among these, 115 (43.5%) had Bethesda III and IV categories. This was a surgical strategy that was adopted in some Swedish centres to perform diagnostic lobectomies plus prophylactic ipsilateral CLND in intermediate FNAC categories during the study period in order to avoid redo ipsilateral CLND along with completion thyroidectomy in cases of malignant final histopathology. However, this practice to our knowledge is currently abandoned in Sweden.

With regards to surgical morbidity, postoperative complications at completion of surgery were 0.5% for RLN palsy and 1% for hypoparathyroidism 6 months postoperatively. The low complication rate of completion surgery in the management of TC in Sweden could be due to the primary diagnostic lobectomy approach in most cases, leaving non-dissected surgical planes of RLN and parathyroid glands on the contralateral side. Nevertheless, the overall postoperative complication rate was higher in primary TT vs. LTT for RLN palsy (4.8% vs. 2.4%; *p* = 0.003) and permanent hypoparathyroidism (6.8% vs. 0.8%; *p* < 0.0001). However, further data analysis revealed that LND, mainly CLND was performed in up to 70% of patients in connection to primary TT in our series, probably illustrating a more aggressive prophylactic approach for CLND in TC during the study inclusion period and prior to the latest ATA recommendations on nodal dissections [14].

Despite that up to 15% of completion thyroidectomies could have been avoided due to absent, inconclusive or misleading FNAC preoperatively, if FNAC had been performed initially or repeatedly as necessary and upfront TT had been applied, completion surgery was not linked with high postoperative complication figures in terms of RLN palsy and permanent hypoparathyroidism. Similarly to our findings, other studies have shown that completion of thyroidectomies following lobectomy has a relatively low rate of complications ranging from 0 to 4.6% for RLN palsy and 0 to 1.5% for other surgical complications [21–23]. Interestingly, postoperative hypoparathyroidism rates for LTT were 8.4% at discharge, 1.3% at 1 month and 0.8% at 6 months postoperatively. The relatively high rate of hypoparathyroidism for LTT at discharge could be due to LTT procedures other than lobectomies, possibly including limited resection of the contralateral lobe that could temporarily endanger and affect the function of the parathyroid glands on both sides. Another possible, although less common, reason could be combined thyroid and parathyroid surgery for thyroid pathology and HPT.

In our series, patients harboring FTC/HTC > 1 cm had a FNAC suggesting FLUS or SFN in 48.5%, suspicion malignancy in 9.1% and malignancy in 15.2%. Though dependent on malignancy rates, lesions with intermediate cytological results i.e. AUS/FLUS or SFN with low-suspicion US features may benefit from clinical observation, whereas nodules with high-suspicion US features may require repeated FNAC, molecular testing and/or surgery [14, 24]. This is indeed a debatable point, as many Bethesda III and IV nodules appear as isoechoic lesions with halo sign (TIRADS 3); hence, surveillance only on the basis of ultrasound features could be of limited clinical value for such lesions. Nevertheless, we did not have a standardized US classification system for the TC cases included herein during the inclusion period, and molecular testing has only been available recently.

Importantly, one of the authors (PL) scrutinized cytology and final histology reports with the aim to assess whether FNAC was obtained from the same nodule described in the histology report. Although this was not always possible, particularly in sub-centimenter TC lesions detected only at final histopathology, we have undertaken sub-analyses for lesions > 1 cm, where FNAC yields higher sensitivity rates, and the lesions are also clinically relevant. Furthermore, in the Swedish clinical practice during the study period, intermediate FNACs, both AUS/FLUS and SFN cytological findings most often led to diagnostic procedures, commonly lobectomies, if there was no other pathology in the contralateral lobe to support TT. Therefore, we have a substantial number of diagnostic procedures in this TC patient cohort, as many as 314 LTTs primarily, following intermediate FNAC findings.

Surprisingly, 24.3% of patients with FTC and/or HTC had malignant or suspicion for malignancy FNAC results. This could be partly attributed to that we included minimally but also widely invasive FTC/HTC cases (sometimes locally advanced with or without distant metastases), and cytologists may have used clinical information to classify these cases as malignant. Other possible explanations could be cytological misdiagnosis or cytological misclassification when recoding cytological reports obtained prior to the Bethesda adoption in Sweden.

With respect to the FNAC impact in surgical strategy for non-WTC, a different surgical approach with TT rather than lobectomy should be applied in MTC and poorly differentiated TC, when suspicion for malignancy and malignant FNAC results are encountered, R0 resection is feasible and non-WTC is suspected [25]. Primary surgery for lymphoma and secondary malignancies as well as for ATC, which is commonly diagnosed in late stages, is not generally recommended [26].

Our study has several limitations. We only focused on TC cases and included patients undergoing surgery from SQRTPA with available histological correlation to FNAC findings after meticulous data scrutinization and nationwide validation. Clinical factors contributing to the decision to refer for surgery with a high clinical suspicion for TC despite the absence of FNAC or in cases of an inconclusive or benign FNAC may have indeed confounded our results. Such factors include suspicion features in US, nodule growth, local compressive symptoms, nodule size, and patient concern or preference. Our dataset also includes non-FNAC considerations leading to surgical referral and final malignant histology. Currently, in the Swedish clinical practice, intermediate FNACs most often lead to diagnostic procedures, commonly lobectomies. In addition, as part of the present cytological data was obtained prior to the widespread Bethesda adoption in Sweden, we recoded these following the Bethesda system, but we did not attempt to distinguish AUS/FLUS and follicular neoplasm categories; therefore, we could not provide separate analyses for Bethesda groups III and IV. Finally, as there was no central cytology or histology review, experience with FNAC specimen procurement and interpretation as well as histology assessment can vary considerably between pathologists/cytologists across different hospitals and might also account for confounding in the present study.

## Conclusions

FNAC results appear to affect thyroidectomy extent in TC as intermediate FNAC categories lead more often to LTT whereas suspicion for malignancy and malignant FNAC categories to TT. Inadequate procedures in non-intermediate FNAC categories necessitating completion surgery are encountered in up to 15% of TC patients subjected to LTT due to absent or misleading FNAC. However, completion thyroidectomy in this setting did not yield significant surgical morbidity. Furthermore, in postoperative surgical morbidity analysis, primary LTT appears to be a safer primary approach compared to TT in respect of RLN palsy and permanent hypoparathyroidism complication rates. Therefore, primary TT should probably be reserved for lesions > 1 cm or even larger with suspicion for malignancy or malignant FNAC.

## Data Availability

Individual patient data included in the present study are available upon request from the corresponding author.
